# Prevalence of papillomavirus infection in women in Ibadan, Nigeria: a population-based study

**DOI:** 10.1038/sj.bjc.6601515

**Published:** 2004-02-03

**Authors:** J O Thomas, R Herrero, A A Omigbodun, K Ojemakinde, I O Ajayi, A Fawole, O Oladepo, J S Smith, A Arslan, N Muñoz, P J F Snijders, C J L M Meijer, S Franceschi

**Affiliations:** 1 College of Medicine, University of Ibadan, PMB 5017, GPO, Ibadan, Nigeria; 2 Proyecto Epidemiologico Guanacaste, Costa Rican Foundation for Health Sciences, PO Box 125-6151, San José, Costa Rica; 3 General Outpatient Department, University College Hospital, Ibadan, Nigeria; 4 International Agency for Research on Cancer, 150, cours Albert Thomas, 69008, France; 5 Department of Pathology, Vrije Universiteit Medical Center, Postbus 7057, NL-1007 MB Amsterdam, The Netherlands

**Keywords:** human papillomavirus, age, sexual habits, education, kola-nut

## Abstract

To investigate the prevalence of and the risk factors for cervical infection with human papillomavirus (HPV) in an inner-city area of Ibadan, Nigeria, we interviewed and obtained a sample of cervical cells from 932 sexually active women aged 15 years or older. A total of 32 different HPV types were identified with an HPV prevalence of 26.3% overall and 24.8% among women without cervical lesions; or age-standardised to the world standard population of 28.3 and 27.3%, respectively. High-risk HPV types predominated, most notably HPV 16, 31, 35 and 58. In all, 33.5% of infections involved more than one HPV type. Unlike most populations studied so far, HPV prevalence was high not only among young women, but also in middle and old age. Single women (odds ratio, OR=2.1; 95% confidence interval, CI=1.1–3.9) and illiterate women (OR=1.7; 95%CI=1.1–2.5) showed increased HPV positivity. Associations were also found with anti-Herpes simplex-2 antibodies (OR=1.6; 95% CI: 1.1–2.1) and with the husband's extramarital relationships (OR=1.6: 95% CI: 1.0–2.6). High prevalence of HPV in all age groups may be a distinctive feature of populations where HPV transmission continues into middle age and cervical cancer incidence is very high.

The prevalence of cervical infection with human papillomavirus (HPV), particularly of high-risk (HR) types that cause cervical cancer (Muñoz *et al*, 2003), varies greatly worldwide. A series of population-based HPV surveys coordinated by the International Agency for Research on Cancer (IARC) has shown a 10-fold variation between some areas in Spain (de Sanjosé *et al*, 2003) and North Vietnam (Anh *et al*, 2003), where HPV prevalence in sexually active women aged 15–65 years was below 2%, and areas in Colombia (Molano *et al*, 2002) and Argentina (Matos *et al*, 2003), where it was 15% or greater. The prevalence of HR HPV types in middle-aged women and the incidence of cervical cancer in the same age group were strongly positively correlated (Franceschi *et al*, 2003a).

The incidence of cervical cancer in sub-Saharan Africa is among the highest worldwide, with age-standardised rates of 35.7 per 100 000 in Bamako, Mali, and 41.7 per 100 000 in Kyadondo, Uganda (Parkin *et al*, 2002), yet only recently has information on the prevalence of cervical HPV infection become available (Serwadda *et al*, 1999; Castellsague *et al*, 2001; Gravitt *et al*, 2002; De Vuyst *et al*, 2003; Xi *et al*, 2003).

Nigeria is the most populous country in Sub-Saharan Africa, with approximately 117 million inhabitants, a life expectancy at birth of 50.6 years in men and 52.6 years in women, child mortality of 159 and 152 per 1000 in males and females, respectively, and per capita total expenditure on health of US$8 per year (http://www.who.int). The annual age-standardised incidence of cervical cancer in Ibadan in 1998–1999 was 19.9 per 100 000 (Parkin *et al*, 2003).

Here, we report the first study from Nigeria of the prevalence of cervical HPV infection.

## MATERIALS AND METHODS

### Study subjects

A survey was carried out in April and May 1999 enumerating the female population of the Idikan community, an inner-city, densely populated area of Ibadan north west local government area, consisting mainly of Muslim people of low socioeconomic level. A total of 2870 women aged 15 years or older were estimated to live in Idikan. After meetings with community representatives, women were contacted up to three times at their home by four female nurses and asked to come to the Idikan Clinic that is run by the Preventive and Social Medicine Department of the University of Ibadan. According to the exclusion criteria of the protocol of IARC HPV surveys, women who: (1) were pregnant; (2) had undergone hysterectomy or conisation; and (3) were physically or mentally unable to undergo an interview and a pelvic examination were not invited to participate in the present study.

Between June 1999 and April 2000, 1390 women (48.4% of those who had been enumerated) came to the Idikan Clinic for an interview and a pelvic examination. Participation was higher among women aged 30 years or older (66.1%) than among younger women (31.9%) on account of the exclusion of pregnant women and low compliance of nonsexually active women. We therefore excluded from the present report 62 self-reported virgins. Furthermore, 14 women who had moved away from Idikan, 36 who refused a pelvic examination, and, despite exclusion criteria, 47 women who were pregnant, six who had been hysterectomised, and 22 women who were physically unable to undergo pelvic examination were also excluded.

The interview was administered in the local language (Yoruba) by four research nurses. The structured questionnaire, similar to the other HPV survey coordinated by IARC (Shin *et al*, 2003), included information on sociodemographic characteristics, smoking and chewing habits, reproductive and menstrual factors, sexual habits of the woman and her husband, and lifetime use of contraceptive methods.

All participants signed informed consent forms according to the recommendations of the IARC and local ethical review committees that had approved the study. When indicated, participating women were treated for minor ailments and given multivitamin supplements.

### Gynaecological examination and specimen collection

A total of 1203 sexually active women underwent a pelvic examination by one of the four female nurses. In order to evaluate the feasibility and validity of different screening methods in Nigeria, two different types of tests were performed: Papanicolau (Pap) smear and visual inspection with acetic acid (VIA). In addition, samples of exfoliated cells from the ectocervix and from the endocervix were collected with two Ayre spatulae and a cytobrush (Cervibrush, CellPath). After the preparation of a Pap smear, the remaining exfoliated cervical cells were placed in 50 ml conic tubes that contained 20 ml of phosphate-buffered saline (PBS). All samples were stored in iceboxes and sent daily to the laboratory of the Pathology Department of the University College Hospital, Ibadan, for processing and storage. Samples of exfoliated cells were centrifuged at 3 000 g and the resulting pellet was diluted in 1 ml of PBS and poured into labelled tubes. All samples were stored at −20°C until shipment on dry ice to IARC.

After taking the smear, 4% freshly prepared acetic acid was generously applied on the cervix using soaked cotton wool swabs and a minute after the cervix was examined to evaluate the presence of: (a) faint acetowhite areas; or (b) dense acetowhite areas (Sankaranarayanan *et al*, 1998).

Participants with abnormal findings at cytological or VIA examination were referred, free of charge, to the Gynaecology Department of the University College Hospital, Ibadan, for colposcopic examination and, if appropriate, biopsy and treatment.

Pap smears were read at the Pathology Department of the University College Hospital, Ibadan, and classified according to the Bethesda system. Pap smear results were used preferentially for study purposes and were available for 1110 women. For 93 additional women, however, a Pap smear was lost (two) or was inadequate (91 women). We, therefore, classified them as abnormal if the VIA had revealed white lesions. Finally, women were also asked to provide 10 ml of blood.

### Human papillomavirus and Herpes simplex-2 (HSV-2) detection techniques

#### Human papillomavirus DNA test

Human papillomavirus testing was performed on exfoliated cervical cells from 1177 sexually active women. To analyse the quality of target DNA for polymerase chain reaction (PCR) testing, cervical specimens were screened with *β*-globin gene-specific primers. In total, 245 samples were found to be *β*-globin negative and, as a result, valid HPV results were available for 932 women.

Human papillomavirus positivity was assessed by general primer-mediated GP5+/6+-PCR and by hybridisation of PCR products in an enzyme immunoassay (EIA) using two HPV oligoprobe cocktails that together detect the following 36 HPV types: HPV 6, 11, 16, 18, 26, 31, 33, 34, 35, 39, 40, 42, 43, 44, 45, 51, 52, 53, 54, 55, 56, 57, 58, 59, 61, 66, 68, 70, 71 (equivalent to CP8061), 72, 73, 81 (equivalent to CP8304), 82 (IS39 and MM4 subtypes), 83 (equivalent to MM7), 84 (equivalent to MM8), and CP 6108. Polymerase chain reaction products that were positive in the EIA were subsequently subjected to further typing by reverse line blot hybridization (RLB). Probes and procedures used for EIA and RLB are described elsewhere (van den Brule *et al*, 2002). In addition, EIA-negative samples were tested by low-stringency Southern blot analysis of PCR products with a cocktail probe of HPV-specific DNA fragments to assess whether HPV types were present that were not represented in EIA oligoprobe cocktails. None of such types was found. Special precautions were taken to minimise false-positive results in the PCR, as has been described elsewhere (Walboomers *et al*, 1999).

HR HPV types for this analysis included HPV types 16, 18, 26, 31, 33, 35, 39, 45, 51, 52, 53, 56, 58, 59, 66, 68, 73, and 82 (Muñoz *et al*, 2003). The group of low-risk (LR) types included all other HPV types. Human papillomavirus infections having more than one HPV type (i.e., multiple HPV infections) were considered as HR if any of the types detected was an HR type.

#### Herpes simplex-2 plasma antibodies

The presence of type-specific plasma IgG antibodies against HSV-2 (anti-HSV-2) was tested using an HSV-2 ELISA assay developed by Focus Technology/formerly MRL (Cypress, California) (Ribes *et al*, 2001). All HSV-2 positive findings were confirmed by means of a second test. Valid HSV-2 results were available from 892 women with valid HPV findings.

### Statistical analysis

Odds ratios (ORs) for HPV positivity and corresponding 95% confidence intervals (CIs) were calculated by means of unconditional, multiple logistic regression equations, adjusted for age (<25; 25–34; 35–44; 45–54; 55–64; ⩾65 years). Variables that showed statistically significant associations with HPV positivity in the age-adjusted analyses were included in the same model and evaluated overall and in separated strata by age group, HR and LR HPV types, and multiplicity of infections. The statistical significance of trends for ORs was assessed by considering the categorical variable as a continuous variable in the logistic model.

## RESULTS

Among the 932 women who had a cytological or VIA examination and valid HPV results, 844 (90.6%) had normal cervical findings. Among the 862 women for whom a Pap smear was available, 19 (2.2%) showed atypical squamous cells of undetermined significance (ASCUS), one showed atypical glandular cells of undetermined significance (AGUS), 37 (4.3%) low-grade intraepithelial lesions, and 15 (1.7%) high-grade intraepithelial lesions. In addition, one *in situ* and one invasive cervical cancer were detected. Among 70 women who were screened only by means of VIA, 12 (17.1%) had faint white lesions and two (2.8%) had dense white lesions. Only nine women reported to have ever had a Pap smear before.

The prevalence of HPV of any type was 26.3, 24.8 and 40.9%, respectively, among women with normal and abnormal cervical findings (Table 1

**Table 1:** Prevalence of 32 HPV types by findings at cytological smear or VIA and overall among 932 women, Ibadan, Nigeria

	**Cytology/VIA**								
	**Normal**			**Abnormal**			**Total**		
**HPV type**	**Single**	**Multiple**	**Total (%)**	**Single**	**Multiple**	**Total (%)**	**Single**	**Multiple**	**Total (%)**
*HPV*−			635 (75.2)			52 (59.1)			687 (73.7)
*HPV*+	141	68	209 (24.8)	22	14	36 (40.9)	163	82	245 (26.3)
*HR HPV*+	93	61	154 (18.3)	17	13	30 (34.1)	110	74	184 (19.7)
*LR HPV*+	48	7	55 (6.5)	5	1	6 (6.8)	53	8	61 (6.6)
									
*High-risk infections*									
16	14	11	25 (3.0)	2	3	5 (5.7)	16	14	30 (3.2)
18	6	8	14 (1.7)	1^a^	3^b^	4 (4.6)	7	11	18 (1.9)
26	0	2	2 (0.2)	1	0	1 (1.1)	1	2	3 (0.3)
31	6	16	22 (2.6)	4^a^	1	5 (5.7)	10	17	27 (2.9)
33	1	4	5 (0.6)	0	0	0	1	4	5 (0.5)
35	12	13	25 (3.0)	1	4	5 (5.7)	13	17	30 (3.2)
39	2	3	5 (0.4)	0	1	1 (1.1)	2	4	6 (0.6)
45	9	9	18 (2.1)	1	0	1 (1.1)	10	9	19 (2.0)
51	6	3	9 (1.1)	1	4	5 (5.7)	7	7	14 (1.5)
52	3	10	13 (1.5)	2	2	4 (4.6)	5	12	17 (1.8)
53	1	1	2 (0.2)	0	0	0	1	1	2 (0.2)
56	6	12	18 (2.1)	2^a^	2	4 (4.6)	8	14	22 (2.4)
58	10	11	21 (2.5)	1	1	2 (2.3)	11	12	23 (2.5)
59	3	2	5 (0.6)	0	0	0	3	2	5 (0.5)
66	12	5	17 (2.0)	0	3	3 (3.4)	12	8	20 (2.2)
68	0	2	2 (0.2)	0	0	0	0	2	2 (0.2)
73	0	4	4 (0.5)	0	0	0	0	4	4 (0.4)
82	2	1	3 (0.4)	1^c^	0	1 (1.1)	3	1	4 (0.4)
Subtotal	93	117	210 (—)	17	24	41 (—)	110	141	251 (—)
*Low-risk infections*									
6	2	1	3 (0.4)	0	5	5 (5.7)	2	6	8 (0.9)
11	0	3	3 (0.4)	0	1	1 (1.1)	0	4	4 (0.4)
34	0	1	1 (0.1)	0	0	0	0	1	1 (0.1)
40	3	5	8 (1.0)	1	0	1 (1.1)	4	5	9 (1.0)
42	20	17	37 (4.4)	0	4	4 (4.6)	20	21	41 (4.4)
43	1	3	4 (0.5)	0	1	1 (1.1)	1	4	5 (0.5)
54	0	2	2 (0.2)	0	0	0	0	2	2 (0.2)
55	0	3	3 (0.4)	0	0	0	0	3	3 (0.3)
70	3	5	8 (1.0)	1	0	1 (1.1)	4	5	9 (1.0)
72	4	6	10 (1.2)	2	0	2 (2.3)	6	6	12 (1.3)
81	9	14	23 (2.7)	1	2	3 (3.4)	10	16	26 (2.8)
83	4	5	9 (1.1)	0	2	2 (2.3)	4	7	11 (1.2)
84	2	0	2 (0.2)	0	0	0	2	0	2 (0.2)
CP6108	0	1	1 (0.1)	0	0	0	0	1	1 (0.1)
Subtotal	48	66	114 (—)	5	15	20 (—)	53	81	134 (—)
Total	141	183	324 (—)	22	39	61 (—)	163	222	385 (—)

HPV=human papillomavirus; VIA=visual inspection with acetic acid.

a It includes one histologically confirmed cervical intraepithelial neoplasm 3.

b It includes one *in situ* squamous cell carcinoma of the cervix.

c It includes one invasive squamous cell carcinoma of the cervix.

). Human papillomavirus prevalence age-standardised to the world population was 28.3% overall, and 27.3% among women without cervical lesions. A total of 82 (8.8% overall, 33.5% of HPV-positive women) had multiple HPV infections and a total of 385 HPV infections with 32 different HPV types were detected. High-risk HPV infections were substantially more frequent (19.7% of all women) than LR HPV infections (6.6%). The most commonly found HPV types, in either single or multiple infections, were HPV 42 (an LR type, 41 women), HPV 16 (30 women), and HPV 35 (30 women), but the type distribution varied by cervical findings. Human papillomavirus 42 was never found in single infections in women with cervical abnormalities. An HR HPV type was found in 34.1% of women with abnormal cervical findings, and in 18.3% of those with normal cervical findings. All three women in whom biopsy showed a cervical intraepithelial lesion grade 3 and two women with *in situ* or invasive cervical carcinomas were HR HPV positive. Combinations of HPV types found in women with multiple HPV infections are given in the appendix.

Figure 1

**Figure 1:**
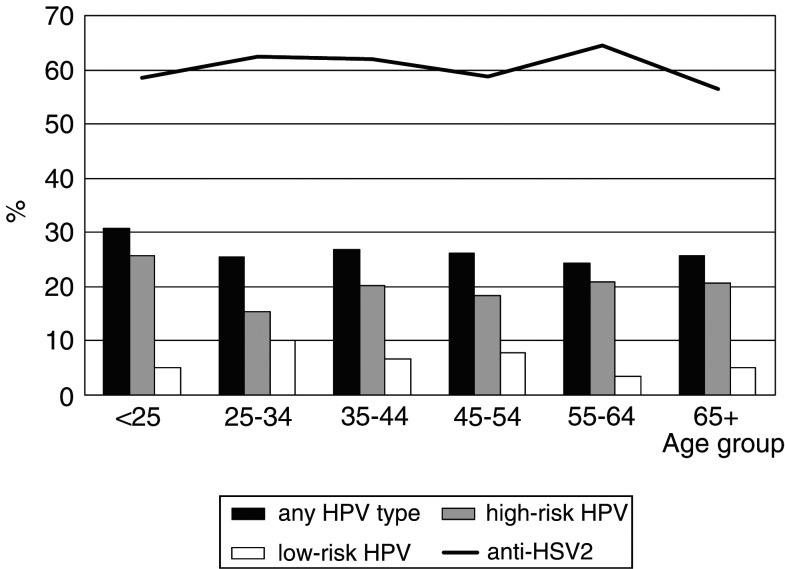
Age-specific prevalence of HPV and anti-HSV-2 antibodies.

shows the age pattern of the prevalence of HPV (any type and HR and LR types separately) and that of anti-HSV-2. Anti-HSV-2 was approximately twice as frequent as HPV positivity in any given age group.

Tables 2

**Table 2:** Odds ratios (ORs) for HPV positivity and corresponding 95% confidence intervals (CIs) according to sociodemographic and reproductive characteristics among 932^a^ women, Ibadan, Nigeria

		**HPV positive**			
	**Number of women**	**N**	**(%)**	**OR^b^**	**95% CI**
*Age (years)*					
<25	120	37	(30.8)	1	
25–34	189	48	(25.4)	0.8	(0.5–1.3)
35–44	134	36	(26.9)	0.8	(0.5–1.4)
45–54	196	51	(26.0)	0.8	(0.5–1.3)
55–64	172	42	(24.4)	0.7	(0.4–1.2)
⩾65	121	31	(25.6)	0.8	(0.4–1.4)
					
*χ*^2^_1_ for trend				0.74;	*P*=0.39
					
*Education*					
Primary or better	495	121	(24.4)	1	
Illiterate	417	117	(28.1)	1.7	(1.1–2.5)
					
*Chewing habit*					
No	600	147	(24.5)	1	
Yes	331	98	(29.6)	1.4	(1.0–1.9)
					
*Marital status*					
Married	687	178	(25.9)	1	
Single	62	25	(40.3)	2.1	(1.1–3.9)
Divorced/widowed	183	42	(23.0)	0.8	(0.5–1.3)
					
*Number of pregnancies*					
0	57	22	(38.6)	1.7	(0.8–3.4)
1–2	134	39	(29.1)	1	
3–4	164	45	(27.4)	0.8	(0.5–1.4)
5–6	230	49	(21.3)	0.6	(0.3–1.0)
⩾7	326	87	(26.7)	0.7	(0.4–1.3)
					
*χ*^2^_1_ for trend				1.96;	*P*=0.16
					
*Age at first pregnancy (years)*					
⩾25	253	69	(27.3)	1	
20–24	479	115	(24.0)	0.8	(0.6–1.2)
⩽19	132	38	(28.8)	1.0	(0.6–1.7)
					
*χ*^2^_1_ for trend				0.02	*P*=0.90

HPV=human papillomavirus.

a Some figures do not add up to the total because a few are missing.

b Adjusted for age.

, 3

**Table 3:** Odds ratios (OR) for HPV positivity and corresponding 95% confidence intervals (CIs) according to indicators of sexual habits and use of contraceptive methods among 932 women,^a^ Ibadan, Nigeria

		**HPV positive**			
	**Number of women**	**N**	**(%)**	**OR^b^**	**95% CI**
*Age at first intercourse (years)*					
⩽20	472	122	(25.8)	1	
18–19	228	60	(26.3)	0.98	(0.7–1.4)
⩽17	231	63	(27.3)	0.99	(0.7–1.5)
					
*χ*^2^_1_ for trend				*0.01*;	*P*=*0.94*
					
*Lifetime sexual partners*					
1	491	118	(24.0)	1	
2	267	76	(28.5)	1.3	(0.9–1.8)
⩾3	170	51	(30.0)	1.4	(0.9–2.0)
					
*χ*^2^_1_ for trend				*3.2*;	*P*=*0.07*
					
*Husband's extramarital sexual relationships*					
No	145	30	(20.7)	1	
Unknown	162	41	(25.3)	1.3	(0.8–2.3)
Yes	625	174	(27.8)	1.6	(1.03–2.6)
					
*Anti-herpes simplex 2 antibodies*					
No	345	74	(21.4)	1	
Yes	547	163	(29.8)	1.6	(1.1–2.1)
					
*Use of hormonal contraceptives*					
Never	805	215	(26.7)	1	
Ever	127	30	(23.6)	0.9	(0.5–1.3)
					
*Use of condoms*					
Never	848	225	(26.5)	1	
Ever	84	20	(23.8)	0.8	(0.5–1.4)
					
*Use of intrauterine device*					
Never	776	199	(25.6)	1	
Ever	156	46	(29.5)	1.3	(0.9–1.9)

HPV=human papillomavirus.

a Some figures do not add up to the total because a few are missing.

b Adjusted for age.

and 4

**Table 4:** Odds ratios (ORs)^a^ and corresponding 95% confidence intervals (CIs) for major risk factors for HPV positivity by age group, HPV type, and multiplicity of infection and overall among 932 women, Ibadan, Nigeria

	**Age**		**HPV type(s)**		**Multiplicity**		
	**<35**	**⩾35**	**Low-risk**	**High-risk**	**Single**	**Multiple**	**Total**
*Age (years)*							
<25	1	—	1	1	1	1	1
25–34	0.8 (0.4–1.3)	—	1.7 (0.7–4.6)	0.6 (0.3–1.01)	1.0 (0.6–1.2)	0.4 (0.2–0.9)	0.8 (0.5–1.3)
35–44	—	1	0.9 (0.3–2.7)	0.6 (0.3–1.1)	0.8 (0.4–1.5)	0.5 (0.2–1.2)	0.6 (0.4–1.1)
45–54	—	1.6 (0.8–2.9)	0.7 (0.2–2.2)	0.5 (0.3–0.9)	0.6 (0.3–1.1)	0.4 (0.2–1.1)	0.5 (0.3–0.9)
55–64	—	1.3 (0.7–2.2)	0.3 (0.1–1.2)	0.5 (0.2–0.9)	0.4 (0.2–0.8)	0.5 (0.2–1.3)	0.4 (0.2–0.8)
⩾65	—	1.0 (0.6–1.8)	0.4 (0.1–1.5)	0.4 (0.2–0.9)	0.5 (0.2–1.00)	0.4 (0.1–1.1)	0.4 (0.2–0.8)
							
*Education*							
Primary or better	1	1	1	1	1	1	1
Illiterate	2.3 (0.8–6.5)	1.6 (1.02–2.6)	2.1 (0.98–4.5)	1.5 (0.96–2.5)	1.7 (1.01–2.7)	1.6 (0.8–3.2)	1.7 (1.1–2.5)
							
*Chewing habit*							
No	1	1	1	1	1	1	1
Yes	0.9 (0.5–1.9)	1.4 (0.9–2.0)	1.5 (0.8–2.6)	1.2 (0.8–1.8)	1.6 (1.06–2.3)	0.8 (0.5–1.4)	1.3 (0.1–1.8)
							
*Husband's extramarital relationships*							
No	1	1	1	1	1	1	1
Yes/uncertain	1.7 (0.9–3.1)	1.1 (0.6–2.1)	1.6 (0.7–3.6)	1.3 (0.8–2.1)	1.4 (0.8–2.5)	1.1 (0.6–2.3)	1.3 (0.8–2.1)
							
*Anti-herpes simplex-2 antibodies*							
No	1	1	1	1	1	1	1
Yes	1.1 (0.7–1.9)	1.8 (1.2–2.6)	1.2 (0.7–2.1)	1.6 (1.1–2.3)	1.3 (0.9–1.9)	2.1 (1.2–3.5)	1.5 (1.1–2.1)

HPV=human papillomavirus.

a Derived from a multiple logistic regression equation that included all the factors listed in the tables.

show the relationship between the HPV positivity and age and the major characteristics of the study women after adjustment for age. Human papillomavirus prevalence was not significantly different in the age groups considered, ranging between 30.8% among women younger than 25 years and 24.4% among those in the 55–64 age range (Table 2). Illiteracy, which was reported by 45.7% of women, was associated with an OR for HPV-positivity of 1.7 (95% CI: 1.1–2.5). Only 1.8% of the study women had ever smoked cigarettes (OR=1.6; 95% CI: 0.6–4.5, data not shown), but 35.6% reported a chewing habit (OR=1.4; 95% CI: 1.0–1.9). In all, 96% of chewers chewed kola-nut, while 12 (3.6%) chewed a mixture of products that, in three women, included tobacco.

Single women, who accounted for 6.7% of our study participants, showed an OR of 2.1 (95% CI: 1.1–3.9), whereas no excess of HPV positivity was found among 43 divorced or 140 widowed women compared to currently married ones. Only 6.3% of women had never been pregnant, whereas 61.0% reported five pregnancies or more. Nulligravidae showed, *vs* women with one or two pregnancies, an OR of 1.7 (95% CI: 0.8–3.4), but the trend in risk by number of pregnancies was not significant. In all, 15.1% of women reported becoming pregnant for the first time before the age 20 years but age at first pregnancy was not significantly associated with HPV positivity (Table 2). Odds ratios for different number of births were very similar to those for number of pregnancies. The OR for HPV positivity among 356 (38.2%) women who reported one abortion or more (mainly miscarriages) was 0.9 (95% CI: 0.7–1.2) (data not shown).

Among indicators of sexual habits (Table 3), age at first intercourse (below 20 years of age in 49.3% of study women) seemed unrelated to HPV prevalence. Two lifetime sexual partners or more were reported by 47.1% of the study women and a direct association of borderline statistical significance was found with HPV positivity (OR for three partners or more *vs* one=1.4, 95% CI: 0.9–2.0). Regular sexual partners (i.e., male sexual partners in relationships that had lasted at least 6 months) and occasional sexual partners were also evaluated separately (data not shown). The OR for two regular sexual partners or more was 1.2 (95% CI: 0.9–1.7). A history of occasional sexual partners (19.1% of study women) was associated with an OR of 1.3 (95% CI: 0.9–1.8). Only 15.6% of study women believed that that their husbands had not had extramarital sexual relationships. The OR for HPV positivity among those women who were certain about their husbands having had an extramarital sexual relationship was 1.6 (95% CI: 1.0–2.6). According to the women, such relationships involved sex-workers only in a minority of cases. The presence of anti-HSV-2 (61.3% of women) was associated with an OR of 1.6 (95% CI: 1.1–2.1). Use of hormonal contraceptives (including 41 women who had used injectable types) and condom was reported by 13.6 and 9.0% of the study women, respectively, and was unrelated to HPV prevalence. Intrauterine devices were used more frequently than any other contraceptive methods and showed an OR of 1.3, of borderline statistical significance (Table 3).

The effect of age group and significant risk factors for HPV positivity in age-adjusted analyses is reassessed in Table 4 by means of multiple logistic regression using all listed variables, overall and separately, in women below and above age 35 years, in HR and LR HPV infections and in single and multiple infections. No significant heterogeneity emerged between the different strata. Among all women, only illiteracy and the presence of anti-HSV-2 retained a significant association in the multivariate analyses. The age pattern of HPV positivity was substantially changed in the multivariate analysis, suggesting that high HPV positivity in older women was accounted for by a greater prevalence of some unfavourable characteristics, chiefly illiteracy, which increased from 5.9% in women below age 25 years to 87.9% in those aged 55 years or older.

## DISCUSSION

The prevalence of HPV positivity of 26.3% found in Ibadan, Nigeria is consistent with previous reports of the elevated prevalence of HPV in women in Sub-Saharan Africa. The age pattern was notable, however, with a modest peak of HPV infections (mainly HR HPV types) among women younger than 25 years and a consistently high prevalence among middle-aged and old women. The prevalence of anti-HSV-2 was also high (61.3%), reaching maximal levels already in women below age of 25 years. Being single and illiterate were the main correlates of HPV positivity among study women.

Previous HPV surveys in sub-Saharan Africa have generally shown relatively high HPV prevalence with some variation, depending on how women were selected, and how HPV was tested for. Using the hybrid capture (HC) assay II, a 17% prevalence of HR HPV types was found in rural Uganda (Serwadda *et al*, 1999), while a 25% prevalence was found among HIV-negative women in Harare, Zimbabwe (Womack *et al*, 2000). Polymerase chain reaction-based assays showed HPV prevalence of 40% in rural Mozambique (Castellsague *et al*, 2001), of 31% in Harare, Zimbabwe (Gravitt *et al*, 2002), of 18% in Dakar and Pikene, Senegal (Xi *et al*, 2003), and of 44% in Nairobi, Kenya (De Vuyst *et al*, 2003). As in most previous studies (Castellsague *et al*, 2001; Gravitt *et al*, 2002; De Vuyst *et al*, 2003), we found that multiple HPV infections were involved in a substantial fraction of HPV-positive women.

Several authors have raised the possibility of certain HPV types being more common in Sub-Saharan African women than elsewhere. HPV 35, for instance, was slightly more common than HPV 16 in Mozambique both in women with normal cytology and in those with HSIL or worse (Castellsague *et al*, 2001). HPV 52 was found slightly more frequently than HPV 16 or HPV 35 in Kenya (De Vuyst *et al*, 2003) and in colposcopically normal women in Zimbabwe (Gravitt *et al*, 2002). In Senegal, HPV 16 and 58 were the most common types overall and in women with cervical lesions (Xi *et al*, 2003). In our study, when the frequency of HPV types both in single and multiple infections were combined, HPV 16 and 35 were the most common HR types, followed by HPV 31, 58, and 56. An LR type, HPV 42, was also common.

Several caveats are relevant to the interpretation of variations in HPV type distribution. As noted by Gravitt *et al* (2002), type-specific HPV prevalence may be influenced by the type of assay used and by the high proportion of multiple HPV infection in certain populations. Furthermore, studies from sub-Saharan Africa have shown variations in the relative ranking of HPV types that are compatible with chance and everywhere the predominance of HPV 16 and 18 rises with the increasing severity of cervical findings (Clifford *et al*, 2003a,2003b). The type-specific distribution of HPV among 799 cervical cancer biopsies from Africa showed that HPV 16 accounted for 50.2% of samples, HPV 18 for 14.1%, and HPV 45 for 7.9% (i.e., a distribution similar to that found worldwide) (Clifford *et al*, 2003b).

The age pattern of HPV prevalence also differs somewhat from one country to another. The predominant pattern includes an early peak, soon after the start of sexual intercourse (Jacobs *et al*, 2000; Kjaer *et al*, 2001), followed by lower levels of HPV positivity in middle age (Jacobs *et al*, 2000; Sellors *et al*, 2000; Molano *et al*, 2002; Anh *et al*, 2003; de Sanjosé *et al*, 2003; Matos *et al*, 2003; Shin *et al*, 2003; Sukvirach *et al*, 2003). U-shaped curves were also found, but the age group with the lowest HPV prevalence was not entirely consistent (e.g., at ages 35–54 years in Costa Rica, (Herrero *et al*, 2000) and 35–44 years in Mexico (Lazcano-Ponce *et al*, 2001)). In three studies from sub-Saharan Africa (Serwadda *et al*, 1999; Castellsague *et al*, 2001; De Vuyst *et al*, 2003), the prevalence of HPV declined with age. In one study (Xi *et al*, 2003) HR, but not LR HPV types, were more frequently detected in older than younger women.

The high prevalence of HPV in middle and old age in Ibadan, Nigeria may have different explanations. A fraction of men and women in Ibadan may continue to have multiple sexual contacts throughout their life and therefore reinfect themselves and their spouses. Women in Ibadan may also have decreased ability to clear HPV infections, possibly due to concomitant genital infections or nutritional deficiencies. We found an age pattern similar to the one in Ibadan in another population at very high-risk for cervical cancer, namely, Chennai, Southern India (Franceschi *et al*, 2003b). Most of the risk factors for HPV positivity we had identified, notably illiteracy (as an indicator of poverty), chewing, and husbands’ extramarital relationships, were more frequent among older than young women.

It is noteworthy that the presence of anti-HSV-2, multiplicity of sexual partners, and husbands’ extramarital sexual relationships were common in Ibadan but that the association of these factors with HPV positivity was relatively weak. As noted with respect to male sexual habits in another high-risk area (Colombia) (Muñoz *et al*, 1996), the effect of variations in individual sexual habits becomes difficult to detect in populations where the background prevalence of sexually transmitted disease exceeds a certain threshold. The number of pregnancies was not significantly associated with HPV positivity. The higher proportion of HPV-positive women among nulligravidae was chiefly due to the fact that 80.7% of them were single (OR for nulligravidae *vs* gravidae, adjusted for marital status=1.0; 95% CI: 0.4–2.7). Finally, the habit of chewing was another correlate of HPV positivity in our study. We have previously reported an association between chewing habit and cervical cancer in Southern India, where paan is chiefly composed of betel leaves, areca nut, and tobacco (Rajkumar *et al*, 2003). In Nigeria, women reported chewing kola-nut almost exclusively (*Cola nitida* and *Cola acuminata*), which is high in caffeine, and serves as a stimulant (Morton 1992).

The potential limitations of our survey include the relatively high proportion of inadequate cervical cell samples and the low participation rate. The relatively high proportion of *β*-globin negativity in the cervical cell samples may be due to problems in the transport or storage of cervical cell samples or to the limited experience of our study nurses in cervical cell collection. High proportions of *β*-globin negative samples have been a problem in other IARC surveys in developing countries (Anh *et al*, 2003; Sukvirach *et al*, 2003). Cytological findings were missing or inadequate in approximately 10% of women and were substituted, in order to stratify HPV prevalence by the presence of cervical abnormalities, by VIA findings. Reliance on VIA, which is known to be less specific than Pap smear (Sankaranarayanan *et al*, 1998), was, however, necessary in fewer than 100 women. Their exclusion would have not altered our findings on HPV prevalence by the presence of cervical lesions. Problems with the preparation, and the classification of Pap smears may explain the relatively low proportion of HPV positivity among women with cervical abnormalities but would not affect our conclusions overall.

With respect to the 390 eligible women who were interviewed but for whom information on HPV or cervical status could not be obtained on account of various technical problems, their distribution by sexual and nonsexual factors was similar to that of women who were included in our final analyses, thus weighing against a clear selection bias. It is also reassuring that the distribution of some of the characteristics investigated (e.g., multiple sexual partners, parity, use of OCs or condoms) was consistent with those reported nationwide by publications of the World Health Organization (http://www.who.int).

Finally, we did not have information on HIV status. The most recent findings from HIV Surveillance in Ibadan showed an HIV prevalence of 3.3% among pregnant women in 2001, 22.6% among female sex workers in 1995, and 6.2% among patients of sexually transmitted disease clinics in 1993 (http://www.unaids.org). Peak infection occurred among women less than 30 years and would, therefore, not explain the high HPV prevalence we found among women of middle and old age.

## Appendix

Combinations of human papillomavirus (HPV) types in 82 women with multiple infections in Ibadan, Nigeria are listed in Table 5

**Table 5:** Combinations of human papillomavirus (HPV) types in 82 women

**HPV type**	**No**	**HPV type**	**No**	**HPV type**	**No**
6,11,**39**	**1**^a^	**18**,42**,52**,81,83	**1**^a^	**39,58**	**1**
6,**18**,42	**1**^a^	**18**,55	**1**	40,42	1
6,**35**	**1**^a^	**18**,72	**1**	40,**52**	**1**
6,**35**,40,**56,73**	**1**	**18,73**	**1**	40,70	1
6,43	1^a^	**18**,81	**1**	42,43	1
6,**66**	**1**^a^	**26,51,56**	**1**	42,**45,56,58**	**1**
11,**18,58**	**1**	**31,35**,43**,52**	**1**	42,55	1
11,**31**34,42	**1**	**31,35,52**,81	**1**	42**,56**	**1**
11**,35,66**,81	**1**	**31,35**,70	**1**	42**,59**	**1**
**16,26,58**	**1**	**31,39,82**	**1**	42,72	1
**16,31,35,51,56**	**1**^a^	**31**,42	**1**	42**,73**	**1**
**16,31,35,52,58**,70,81	**1**	**31**,42,72,81	**1**	42,81	1
**16,31,51**	**1**	**31**,42,81,83	**1**	43,**56**,81	**1**
**16**,40,54,**58,66**	**1**	**31,56,58**	**1**	**45,52**,81	**1**
**16**,42,72	**1**	**31,59**	**1**	**45,53**	**1**
**16**,42,83	**1**^a^	**31**,81	**1**	**45**,81	**1**
**16,45**	**1**	**31**,81,CP6108	**1**	**51,52,56**	**1**^a^
**16,52**	**1**	**33,45**	**2**	**51,66**	**1**^a^
**16,52**,54**,66**	**1**	**33,52**	**1**	**52**,72	**1**
**16,52**,55	**1**	**33,56**	**1**	**56,58**	**1**
**16,58,66**,70,83	**1**	**35**,42	**2**^*^	**56**,81	**1**
**16,66**	**1**^a^	**35**,42**,56,58**	**1**	**56**,83	**1**
**16**,83	**1**	**35,45**	**1**	**58**,72	**1**
**18,31**	**1**	**35,51**	**1**	**58**,81	**1**^a^
**18,31**,42	**1**	**35,66**	**1**	**68**,81	**1**
**18,35,51**	**1**^a^	**35,73**	**1**	70,83	1
**18,35,56,68**	**1**	**39,45**	**1**		

High-risk types in bold face.

a One women with abnormal cervical findings.

.
